# Quantification and classification of potassium and calcium disorders with the electrocardiogram: What do clinical studies, modeling, and reconstruction tell us?

**DOI:** 10.1063/5.0018504

**Published:** 2020-10-02

**Authors:** N. Pilia, S. Severi, J. G. Raimann, S. Genovesi, O. Dössel, P. Kotanko, C. Corsi, A. Loewe

**Affiliations:** 1 Institute of Biomedical Engineering, Karlsruhe Institute of Technology (KIT), 76131 Karlsruhe, Germany; 2 Department of Electrical, Electronic, and Information Engineering “Guglielmo Marconi,” University of Bologna, 47522 Cesena, Italy; 3 Renal Research Institute, New York, New York 10065, USA; 4 Department of Medicine and Surgery, University of Milan-Bicocca, 20100 Milan, Italy; 5 Icahn School of Medicine at Mount Sinai, New York, New York 10029, USA

## Abstract

Diseases caused by alterations of ionic concentrations are frequently observed challenges and play an important role in clinical practice. The clinically established method for the diagnosis of electrolyte concentration imbalance is blood tests. A rapid and non-invasive point-of-care method is yet needed. The electrocardiogram (ECG) could meet this need and becomes an established diagnostic tool allowing home monitoring of the electrolyte concentration also by wearable devices. In this review, we present the current state of potassium and calcium concentration monitoring using the ECG and summarize results from previous work. Selected clinical studies are presented, supporting or questioning the use of the ECG for the monitoring of electrolyte concentration imbalances. Differences in the findings from automatic monitoring studies are discussed, and current studies utilizing machine learning are presented demonstrating the potential of the deep learning approach. Furthermore, we demonstrate the potential of computational modeling approaches to gain insight into the mechanisms of relevant clinical findings and as a tool to obtain synthetic data for methodical improvements in monitoring approaches.

## I. INTRODUCTION AND BACKGROUND

Impairment of potassium homeostasis is common and can be iatrogenic, for example, caused by the use of diuretic drugs or due to diseases, e.g., chronic kidney disease (CKD), myocardial infarction, etc.^1,2^ Similar to potassium, the impairment of other electrolyte concentrations is also highly relevant in clinical practice,^1^ and for all use cases, a rapid non-invasive point-of-care (POC) diagnostic tool is desirable. This could allow for early diagnosis and improvement of strategies to optimize treatment and consequently improve patient outcomes in emergency settings.^1^ Apart from an emergency setting, (clinical) studies could also benefit from a non-invasive, rapid, and cost-efficient tool for diagnosis of electrolyte imbalance. Interesting examples in this context are studies in haemodialysis (HD) patients. This patient population is known to suffer from a 14-fold increased risk of dying from sudden cardiac death (SCD) compared to patients with cardiovascular diseases without known renal impairment.^3^ The reasons for this elevated mortality risk are multi-factorial and not entirely clearly distinguishable, but factors such as fluid overload, long-standing elevated blood pressure and cardiac strain, and electrolyte impairments are in large part responsible. Complications such as tachy- and bradycardia are frequently observed, not only during HD session but also during the inter-dialytic intervals, in particular, during the first short interdialytic interval and at the end of the long interdialytic interval when patients are at home.^4–6^ The hypothesis of alterations in plasma electrolyte concentrations being at least partly responsible for the increased death rate stands confirmed by much of the published data.^7–10^ Since currently only blood sample assessment provides electrolyte concentration values, a non-invasive diagnosis technique applicable at the POC, at home (or even retrospectively with pre-existing measurements), could allow for insight into the connection between arrhythmia, including SCD, and the alterations of plasma electrolyte concentrations.^11^ If the electrocardiogram (ECG) could be utilized for plasma electrolyte concentration monitoring, a continuous or retrospective assessment of the plasma electrolyte concentration values could also be possible. This idea is linked to the strong dependency of plasma electrolytes (mainly potassium and calcium) that are involved in the genesis of cardiomyocyte action potentials (APs) and ECG pattern changes.^12^ Besides the usefulness in electrolyte monitoring, the ease-of-use and ubiquitous availability of the ECG as a measuring technique are of note. ECG is commonly employed and considered well established in other diagnostic domains, where it is, for example, common practice to monitor patients at home using Holter ECG measurements. Moreover, a trend toward home monitoring with smart devices, notably many of those with an ECG monitor functionality, substantiates the attractiveness of this diagnostic monitoring technique, as pioneered by studies successfully using the ECG for the estimation of electrolyte concentrations.^13–15^ Generally, HD patients are not only of special interest due to their increased risk of SCD but also as in this population, ECG data with concomitant assessments of electrolyte concentrations seem relatively easy to obtain. Nevertheless, we should keep in mind that the standard and benchmark method for concentration measurements, i.e., blood testing, is associated with considerable uncertainty as well.^16^ Until now, we only considered electrolyte concentration estimation without more precisely defining what estimation implies in this field. Estimation of plasma electrolyte concentrations describes the application of either regression or classification to detect an impaired electrolyte homeostasis. When performing regression, we are interested in the exact plasma electrolyte concentration value, i.e., we are quantifying. We can measure mean errors and standard deviation of errors in mmol/l for every used estimation method. With classification, however, we are only interested in a diagnosis; we are not (e.g., “hyperkalemia”), or only roughly (e.g., “severe hyperkalemia”), quantifying the concentration. In the latter case, the greater interest is in sensitivity and specificity rather than performance indices. We will discuss both approaches and provide typical applications for each.

**FIG. 1.**
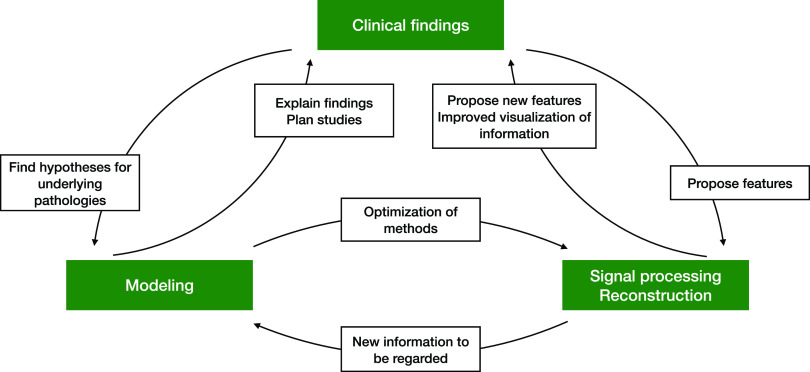
Each section of this work can be attributed to at least one of the topics in the green boxes. Although they are separated in this diagram, they are closely connected and depend on each other. That is why collaboration between the fields is of paramount importance.

This review article is written with a particular focus on potassium and calcium concentration imbalances, mainly because these two ions have been considered highly relevant in recent clinical, modeling, and reconstruction studies. The effects of these ion concentrations on ECG patterns have been reviewed previously.^17–23^ We provide a focus on how computational models can be used to explain and discuss theories on the underlying mechanisms of ECG changes in relationship to electrolyte disorders. Moreover, we summarize how findings from clinical and modeling studies were used to reconstruct potassium concentrations from the ECG (Fig. 1).

### A. Structure of this article

In Sec. II, we focus on recent studies with more than 60 subjects investigating the incidence of ECG feature changes caused by concentration shifts. For most studies, the ECGs were evaluated manually. We also consider studies proposing new feature extraction approaches that will potentially be relevant for automatic reconstruction in the future. In Sec. III, we summarize how modeling can help to understand certain questions and effects that are observed clinically. In Sec. IV, the actual assessment of electrolyte concentrations from ECG analysis is reviewed. First, it is discussed if automatic reconstruction is feasible. Afterward, studies that attempted to reconstruct values or to classify the concentration disorder manually are presented. Further, we report on recent advances in automatic reconstruction and classification. In the final section, we point out open questions and propose further steps to take from a macroscopic view of the field. The general structure is shown in Fig. 1.

## II. CLINICAL IMPACT OF ECG CHANGES CAUSED BY ELECTROLYTE PLASMA CONCENTRATION MODIFICATIONS

**FIG. 2.**
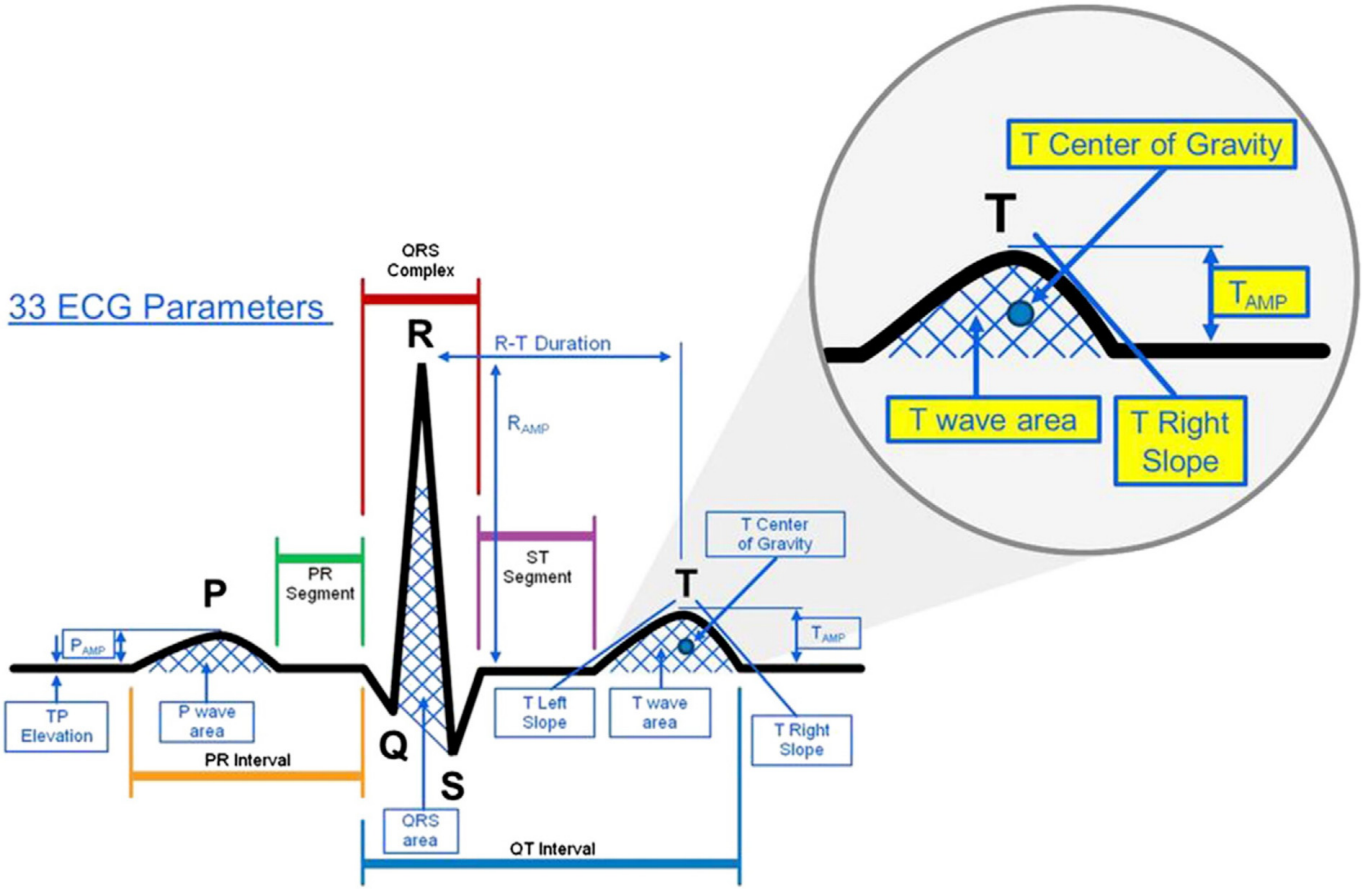
Typical ECG features evaluated to determine concentration changes. The figure visualizes only a subset of 33 features that were analyzed in this work. Reprinted with permission from Dillon *et al.*, J. Electrocardiol. **48**(1), 12–18 (2015). Copyright 2015, Elsevier.

Over the last few years, many clinical publications have been presenting ECG changes caused by electrolyte imbalances (partly) observed for decades.^17–23^ Typical changes caused by potassium and calcium imbalances are reported for the P wave, QRS complex, ST segment, T wave, and possible U waves (some exemplary features capturing these changes are shown in Fig. 2). However, the ECG changes resulting from these studies, the incidence of these changes, and theories behind them are still discussed controversially as described in the Subsection II A. Apart from the single beat features, non-obvious rhythmical (meta) features such as heart rate variability (HRV) parameters are studied as well. In this section, we do not aim to summarize all the known ECG changes regarding morphological and temporal features due to electrolyte concentration changes as these have been reviewed elsewhere.^17–23^ We rather focus on the recent discussion regarding the incidence of feature changes in large clinical studies and the approaches for capturing features apart from the common temporal and morphological ones as well as on the special interest in the QT interval. We also include studies comparing the pre- and post-dialysis state of a patient because of the changes in potassium concentrations during most of the treatments.

### A. ECG changes as a diagnostic tool

Over the last few years, studies have discussed the incidence of electrolyte change-induced ECG abnormalities and the idea of using the ECG as a monitoring tool to prevent adverse outcomes. Works considered in this section comprise more than 60 patients each and are based on manual feature detection.

In a recent meta-analysis, Noordam *et al.* evaluated five ECG intervals with respect to their dependency (among others) on potassium and calcium plasma concentrations:^24^ QT, QRS, PR, RR, and JT interval (being the difference between QT and QRS). The study included 153 014 individuals from 33 studies. They showed that potassium and calcium plasma concentrations influenced all these ECG features; however, in the case of calcium concentration variation, RR, QT, and JT intervals were influenced the strongest. Regarding potassium concentration variations, QRS interval changes were the only being more prominent than those for calcium. Both ions influenced all ECG features, which will be relevant for estimation. As the authors admit, causality was not attempted to be determined in their meta-analysis. Further drawbacks were discussed by Olshansky in a letter.^25^ They state that with such huge numbers of analyzed patients, which can be seen as a strength of this study at first sight, even small changes in the features become significant. This is in accordance with conclusions in the work of Khalilzadeh and Tasci.^26^ When interpreting the results, one needs to bear in mind that even if significant, small changes (the found *β* coefficient quantifying the influence of potassium variations on the QT interval was approximately 2 ms/mmol, for calcium variations 15–20 ms/mmol, both significant) might be clinically irrelevant since it will be impossible to capture them with sufficient accuracy and robustness.

Apart from interval changes, there are typical morphological features described in the literature, especially in the case of potassium variation. An *et al.* studied 923 patients with severe hyperkalemia.^27^ Spiked, tall T waves with an increase in T wave amplitude as well as sine wave-shaped QRS complexes were reported in hyperkalemic patients. However, these changes only occurred in 36.7% of the patients. This was little surprising as the most common underlying clinical condition was chronic kidney disease (CKD); it is well known that patients with CKD often present with a reduced incidence of ECG modifications during elevated plasma potassium concentrations as reported in a study of 74 subjects by Aslam *et al.*^28^ Similar findings were reported by Montague *et al.*,^29^ where in 90 patients (48% with chronic renal failure), no diagnostic threshold could be established for changes in T wave amplitude and T wave peaking as a means to distinguish between normo- and hyperkalemia. Of note, however, T wave peaking was measured subjectively, and thus, intra- and inter-observer variability might have affected the results. Consistently, Yoon *et al.* reported an analysis of 124 238 ECGs with a corresponding concentration measurement within five minutes from ECG acquisition^30^ and concluded that manual ECG diagnosis was not capable of predicting the serum potassium level. Notably, they used T wave amplitude, T wave right slope, and the amplitude normalized slope for reconstruction (Sec. IV C) and only considered measurements with consistent annotation from two independent experts.

Finally, a study comprising 62 HD patients, conducted by Astan *et al.*,^31^ reports an increase in the following parameters: P wave amplitude, QRS amplitude, QRS duration, QTc dispersion, sum of amplitudes of the S peak in V1 and of the R peak in V5, and total QRS amplitude from pre- to post-HD session, where serum potassium is significantly reduced in a majority of patients. Additionally, a decrease in the T wave amplitude and QTc duration was shown. In this study, P and QRS amplitude changes were hypothesized to be caused by a volume loss, which lead to an impedance increase and, therefore, to an increase in both amplitudes. This theory was substantiated on the one hand by the fact that patients with edema had a lower amplitude change. On the other hand, Kinoshita *et al.* hypothesized that while QRS changes could also be caused by the extracellular volume change,^32^ T wave amplitude decreased after HD rather due to other factors compared to a direct effect of the volume loss, e.g., a concentration shift.

The hypotheses of Astan and Kinoshita suggest reasons for why the use of the ECG for concentration monitoring in a clinical setting can create equivocal and sometimes even contradictory results. Cross-dependencies from other effects may impede the adequate detection of concentration changes with the ECG. Whereas some authors of the presented studies took a more skeptical stand toward the interpretation of ECG changes,^31,32^ even further complicated by inter-annotator variability, mainly, the study reported by Noordam *et al.*^24^ promoted a possible use case for the application of ECGs to monitor electrolyte changes. Picking up the problem of inter-annotator variability, the study by Yoon *et al.*^30^ tried to increase the objectivity of ECG assessments by incorporating rating by two annotators, morphological features like the slope were only determined manually and are, therefore, prone to intra- and inter-annotator variability. A clear view on the problem of ECG-based reconstruction of ion concentrations cannot be conveyed from the presented studies as many of them suffer from drawbacks regarding objectivity and reproducibility, which is, of course, hard to guarantee in a clinical context. However, one should keep in mind that semi-automatic or fully automatic approaches can overcome drawbacks from these studies at least in terms of consistency, robustness, transparency, and reproducibility.

### B. ECG features related to cardiac rhythm

The feature extraction methods from the studies mentioned before comprised the analysis of single waves regarding their morphology. Apart from that, it is also possible to analyze the time series of beats yielding rhythmical features. This was done by Lerma *et al.*,^33^ who employed Poincaré plot analysis to circumvent the drawbacks of classical HRV features capturing only the magnitude of variations. Their study comprised ten healthy subjects and ten HD patients. This analysis technique reveals non-linear structures in the time series. Exactly, these structures were found to be reduced in HD patients after dialysis compared to the pre-dialysis state. The loss of this non-linear behavior of HRV could be interpreted as a reduced capability of the patients to respond to changed conditions. Risk stratification using HRV was reported by Chen *et al.*^34^ They compared the dynamics of HRV parameters before and after HD in 182 patients. In a follow-up, they repeated this comparison and analyzed if the HRV change from pre- to post-dialysis is associated with the risk of death based on the data of 29 deaths that occurred during the follow-up period. Four HRV parameters [very low frequency band power (VLF), low frequency band power divided by total power VLF in percent (LF%), high frequency band power divided total power VLF in percent (HF%), and low frequency band power divided by high frequency band power (LF/HF)] significantly changed comparing pre- and post-HD settings. For one parameter (ΔLF%, pre- vs post-HD session), they were able to establish a prediction for total and cardiovascular mortality. To overcome the drawback of HRV being dependent on several beats, González *et al.*^35^ proposed using recurrence plot analysis to capture changes also in short-time ECGs. They were able to show in a study with 19 HD patients and 20 healthy subjects that HRV dynamics were different between these two groups.

All these studies indicate that rhythmical features change between pre- and post-HD settings. Comparing pre- and post-HD settings, the plasma electrolyte concentration usually changes, too. The connection between rhythm and a change of plasma electrolyte concentrations was also reported by El-Sherif and Turitto.^23^ Therefore, rhythmical features are worth to be considered during estimation as they might deliver additional information about the dynamics of the rhythm. To this point, however, the parameters discussed in this section were not used in the studies presented in Sec. IV and still need to be proven to deliver an additional benefit to the problem of concentration estimation in clinical practice.

### C. QT interval changes and electrolyte concentration imbalances

Generally, the QT interval is of special interest for researchers and clinicians since a QT interval prolongation is known to correlate with arrhythmia and SCD.^3,36^ As stated before, the SCD rate is increased in HD patients suffering from frequent electrolyte plasma concentration imbalances and the question remains if there is a direct connection between concentration changes, SCD occurrence, and changes of the QT interval.

Kim *et al.* screened 330 HD patients in the pre-dialysis phase and showed that the QT adjusted for heart rate (QTc) prolongation, observed in 47% of the patients, was related to the changed calcium and potassium plasma concentration.^37^ A one year follow-up examination revealed that 31% of patients had persistent QTc prolongation and the association with the changed plasma electrolyte concentrations of calcium and potassium still existed. The authors concluded that these concentrations should be closely monitored to allow us to compensate the risk for QTc prolongation. If the QT interval change was prevented, arrhythmia risk could be, too. Consequently, the risk for SCD would be reduced as well.^36,38^

The influence of HD vintage on the QTc interval was studied by Matsumoto *et al.*^39^ in ECGs of 102 HD patients and a control group of 68 age-matched patients, analyzed at HD initiation and one, four, and seven years later. The average QTc after four and seven years in the HD group was significantly longer than in the first year after HD initiation, whereas in the control group, such a relation could not be observed. This fact substantiates the need for screening QTc intervals in HD patients as already suggested by Kim *et al.* Additionally, the corrected calcium level and presence of diabetes were associated with longer QTc, which is of particular interest for calcium concentration estimation and for SCD risk stratification. In a study comprising 29 patients, the RR-QT ratio varied during HD.^40^ Moreover, changes in the plasma calcium concentration during HD sessions were inversely correlated with the QTc duration. The same authors showed that different combinations of potassium and calcium concentrations in the dialysate lead to different modifications of the QT interval duration.^41^ This finding was also confirmed by Severi *et al.*^42^

Apart from QT and QTc, QTc dispersion was considered in the study by Cupisti *et al.* comprising ten uremic patients.^43^ To show the influence of the potassium removal rate on QTc dispersion, dialysates and ultrafiltration rates were changed during HD sessions. The authors concluded that QTc dispersion increased with lower potassium plasma levels in connection with the ultrafiltration rate. Thus, not only the absolute concentration seems to be relevant but also the rate of change.

Although most clinical studies focus on HD, peritoneal dialysis (PD) also showed an influence on QTc similar to those that occur during an HD session.^44^ In 15 PD patients, two manual PD exchanges were performed with different calcium dialysate concentrations. The potassium concentration was inversely correlated with QTc changes (r = −0.81). Computer models confirmed this finding. However, the changes in the QT interval observed during a PD exchange were less important than those observed during an HD session. The authors conclude: as significant QT interval changes could be correlated with an increased risk of arrhythmia, PD should be preferred to HD in end-stage renal disease (ESRD) patients at high cardiovascular risk.

QT, QTc, and QT dispersion seem to be closely related to potassium and calcium plasma concentrations or, at least for potassium, their rate of change, which can be influenced in HD patients also by the dialysate potassium concentration. As QT interval changes are associated with SCD, an optimized monitoring of plasma electrolyte concentrations could be a tool to fight the high mortality rate in HD patients with electrolyte imbalance. Regarding the actual ECG-based concentration monitoring, the relation between the QT interval and the ionic concentration could be exploited. Until today, no study directly reconstructed potassium concentrations from the changes in the QT interval, which could be connected to the many other influencing factors on the QT interval (e.g., HD vintage). Nevertheless, QT changes were shown to be sensitive to concentration changes, leading to an additional eligible feature for automatic reconstruction provided that other influencing factors can be corrected.

### D. Model parameters as robust ECG-derived features

On the way to automatic feature evaluation, robustness and precision are of greatest importance. However, at all levels, from beat annotation to feature calculation, errors are possible. Specifically, ECG wave boundaries (beginning and end) and the way of capturing morphological variation are not always as robust as expected. Methods trying to solve the problem of detecting T wave boundaries and features have been sought: Rodrigues *et al.*, for example, utilized the parameters of model functions for the quantification of T wave morphology changes.^45^ To account for the asymmetrical shape of T waves, the sum of a mirrored lognormal curve and a Gaussian function was fitted, and so the analysis of the T wave results in the computation of two parameters for one functional term. In this way, one takes advantage of the fact that, by fitting a predefined curve, *a priori* knowledge about the shape of the T wave can be utilized. Furthermore, it reduces the variations to the expected shape of the wave, which renders the evaluations more robust. All the four parameters, which are parameterizing the two model functions, were then used to assess T wave peakedness combining them to one parameter, which was shown to qualitatively change with changes in the serum potassium concentration. However, no quantification, e.g., by calculating correlation values, was conducted. Very similarly, the method of T wave warping was recently presented by Palmieri *et al.*^46^ The idea behind it can be summarized as follows: two waves are different if a lot of warping of one is needed to fit the second. The measured degree of warping, i.e., the difference of the waves, with respect to a patient-specific mean wave can be correlated with the serum potassium concentration change. This led to median correlation values of 0.9 (interquartile range 0.3) in a study with twelve patients.

Both approaches presented in this section might be a great step toward robust feature extraction having the potential to be applicable for serum potassium concentration estimation from the ECG as they were explicitly developed for this purpose. Nonetheless, the methods need to be validated in larger cohorts to be finally translated to clinical practice.

## III. MODELING ELECTROLYTE DISORDERS

As described in Sec. II, patients exhibit heterogeneous ECG changes in response to electrolyte concentration changes. Large-scale studies are required to draw robust conclusions on the reliability of the ECG as a tool for concentration estimation. However, it is impossible to exclude every confounding factor to fully understand underlying causality. Computational modeling, however, offers a possibility to minimize the effect of uncontrollable influences. Here, we have the opportunity to adjust analyses for parameters and observe their specific influences under controlled conditions. Furthermore, modeling may enable us to non-invasively study influences of diseases, anatomical variations, etc. One should keep in mind that published models were built for a certain purpose and are not always applicable to new problems. They usually need to be fitted to the application. The proposed models for plasma electrolyte concentration change simulation are summarized in Subsection III A. We focus mainly on the results obtained from models regarding electrolyte concentration-induced modifications in the ECG. Works with a focus on the actual optimization process, as, e.g., the work of Carro *et al.*,^47^ are not discussed. In Subsection III B, we present application examples of modeling techniques to optimize signal processing and reconstruction methods in the field of plasma electrolyte concentration estimation.

### A. Modeling as a means to understand phenomena

Modeling can be used to confirm and mechanistically underpin empirical findings, as it was done in the aforementioned studies by Severi *et al.*^42^ and Genovesi *et al.*^44^ In both works, a ventricular action potential (AP) model (a modified ten Tusscher *et al.*^48^ and the more recent O'Hara *et al.* model,^49^ respectively) was used to capture cardiac repolarization changes caused by dialysis observed as QT interval changes in the ECG of a patient cohort. In both cases, the AP duration changed when electrolyte concentrations were changed according to the alterations observed in patients. The simulation results were in line with the clinically observed QT changes and were used to substantiate the findings and hypotheses of the cause for the observed phenomena. Modeling can also help to investigate the question of contradictory inter-patient feature changes for an electrolyte disorder. The influence of transmural heterogeneity on T wave features was shown by Bukhari *et al.*^50^ The group quantified how surface ECG features present for 19 different transmural distributions of endo-, mid-, and epi-cardial cells. The authors report that a different distribution alone can lead to differences in the features. This could partly explain different findings in study populations as we expect variability of spatial ion channel distribution in different patients.^51^ As a last example, modeling can be used for early hypothesis testing. Both tachy- and bradycardia are observed in the interdialytic phase of HD patients. As Loewe *et al.* showed with a human sinus node cell model, hypocalcemia has a marked effect on the excitation rate of the sinus node.^10^ With the decreasing calcium concentration, the heart rate decreased, too. This offers a possible explanation for the observed bradycardia sudden death events in HD patients. As pronounced inter-species differences in the response to hypocalcemia exist,^52^ a human study is strongly recommended. Here again, ECG-based electrolyte monitoring could facilitate such studies.

From the presented studies, we can appreciate how modeling can be utilized to gain mechanistic insight into clinical findings, validate them, and setup new hypotheses. We consider modeling and simulating a very promising and valuable approach to follow when evaluating studies and planning future experiments. This is also of special interest for ECG-based concentration estimation. Potential features can be found and explained. As well, cross-dependencies of features (e.g., as already explained with the QT interval) with other diseases can easily be identified and in a further step corrected during concentration estimation.

### B. Modeling to optimize methods

Modeling, however, can be used not only for the retrospective analysis of findings but also to generate hypotheses. The model of O'Hara *et al.* was used by Kharche *et al.* to study the influence of the extracellular potassium concentration on APs and on a pseudo-ECG.^53^ However, appropriate AP changes caused by calcium modifications are not reproducible with the model since calcium-dependent inactivation of the L-type calcium channel is not strong enough.^54^ Severi *et al.* found that many of the most popular cardiac cellular models were not able to model these calcium concentration-induced AP changes.^55^ Thus, Bartolucci *et al.* proposed a novel model of human ventricular AP to account for this problem.^56^ L-type calcium channels, exchangers, diffusion, and further currents were refined, and the adapted model faithfully reproduces AP shortening caused upon increased calcium concentration. Himeno *et al.* proposed a cell model with refined calcium handling.^57^ In contrast to the model of O'Hara *et al.*, which served as the basis, the model of Himeno *et al.* predicts AP shortening based on the extracellular calcium concentration increase,^57^ which is in line with clinical and experimental findings and particularly important for studying the influence of a calcium concentration change. To reproduce the repolarization change on the ECG level appropriately, model extensions were required since the proposed method is single cell only. Loewe *et al.* proposed a heterogeneous formulation of the model for the simulation of body surface ECGs.^58^ The introduction of epi- and M-formulations and an apico-basal gradient for the *I_Ks_* current (as described by Keller *et al.*^59^) paved the way for ECG simulation and a realistic change of the QT interval dependent on the calcium concentration change. A more detailed analysis of the changes was done by Hernández-Mesa *et al.* regarding AP and ECG features.^60^ Here, the authors captured changes of five AP and twelve ECG features, all calculated automatically. Several temporal and morphological AP and ECG features showed changes, e.g., an increase in the calcium concentrations resulted in an AP duration decrease (as described in Ref. 61) and an ST-segment amplitude increase in the surface ECG (as clinically observed in Refs. 61–63). These changes could be a basis for ion concentration reconstruction. The simulated ECGs by Hernández-Mesa *et al.* can be used not only for assessing the concentration-induced changes in AP and ECG but also to improve signal processing methods. Hernández-Mesa *et al.* studied the optimal ECG leads for feature extraction regarding a following reconstruction.^64^ During automatic analysis, this is usually overcome by using lead transforms like principal component analysis (PCA). For two simple exemplary reconstruction methods, the choice of a particular standard lead or transformed lead influenced the concentration estimation performance for calcium and potassium. Standard leads were performing well only in the case of noise-free signals. When noise was added to the signals, PCA improved the result dramatically due to its filtering properties. Lead transform also influenced the feature concentration relationship, e.g., changing it from nearly linear (good results with a linear fit) to a non-linear dependence (poor results with a linear fit).

The presented examples show the quality and potential of modeling being an enabler for methodological improvement, e.g., regarding feature determination, preprocessing, or finding the optimal work-flow for concentration estimation. Using those optimized methods, the accuracy of concentration estimation methods can further be improved.

## IV. QUANTIFICATION AND CLASSIFICATION OF CONCENTRATION DISORDERS FROM THE ECG

After clinical trials based on manual feature extraction presented in Sec. II raised doubts whether the ECG is an appropriate readout for concentration monitoring, this needs to be re-evaluated. Works on evaluating the feasibility of automatic concentration estimation will be presented in Subsection IV A. The reconstruction of calcium was only performed on simulated data so far. These proof-of-concept studies are described in Subsection IV A. Section IV B presents earlier attempts using manual or semi-automatic potassium estimation approaches, which is particularly interesting to be able to benchmark the automatic approaches that are presented in Sec. IV C.

### A. Feasibility and limits of automatic reconstruction

Extensive feature analysis was performed by Dillon *et al.* to assess the potential of the ECG for plasma electrolyte concentration reconstruction.^65^ The researchers investigated whether small changes in the plasma potassium concentrations were quantifiable in available ECG measurements using one baseline measurement per patient and whether estimation could be applicable in a clinical context. 12-lead ECG templates from twelve HD patients were used to calculate the Akaike Information Criterion (AIC) for ranking fitted linear mixed models utilizing five features across all leads. The features were T downslope, T amplitude, the center of gravity of the T wave and of the last fourth of the T wave, and the ratio between the T wave and R peak amplitude. The lowest overall AIC rank (i.e., showing the best feature concentration relation) was found in V4. However, as the authors state, leads were not statistically different. Across all features, the T downslope was the parameter showing the highest dependence on the plasma potassium concentration, followed by the two center of gravity features. Both amplitude features provided a significantly different (comparing different patients) regression line slope, meaning that the remaining features might be more appropriate for a patient-independent fitting. By applying fuzzy clustering to a reduced set of PCA coefficients of each ECG, Dillon *et al.* evaluated the ECG variations independent of calculated features. The authors found 0.2 mmol/l as the smallest detectable change.

The issue of calcium concentration estimation was addressed in a multi-scale simulation study^66^ based on the cellular model of Himeno *et al.*,^57^ extending a previous work^67^ based on the ten models of ten Tusscher and Panfilov,^68^ with the former being a more realistic model regarding the intracellular calcium handling. 71 simulated 12-lead ECGs with different calcium and potassium concentrations were used to consider cross-dependencies between both electrolyte changes. Following a lead reduction technique transforming the eight independent standard ECG leads into the direction of maximum T wave amplitude, ten single beat features were extracted and subsequently reduced using canonical correlation analysis: T amplitude, T upslope, the energy of the first half of the T wave (normalized to the energy of the whole T wave) and the ratio between R peak energy (signal energy of the R peak) and R peak amplitude, and the ST center (measuring an ST change) were identified as the five most linearly independent features. To prevent overfitting, an artificial neural network (ANN) with regularization and only four neurons was used for reconstruction being able to fit highly non-linear relationships in arbitrary dimensional spaces. Using the selected features, the potassium estimation errors were −0.01 ± 0.14 mmol/l in the noise-free case and −0.03 ± 0.46 mmol/l in the case of distorted simulated ECG beats (30 dB signal-to-noise ratio). For calcium, the errors were 0.01 ± 0.11 mmol/l and 0.02 ± 0.17 mmol/l, respectively. To the best of our knowledge, this is the first study representing a proof-of-concept of calcium concentration reconstruction.

The presented studies underline feasibility and limits of the ECG-based concentration estimation. For potassium concentration estimation, a change as small as 0.2 mmol/l seems to be detectable in recorded ECGs. For calcium concentration estimation, only simulated data were evaluated, suggesting the general feasibility of the method. Nevertheless, clinical ECGs need to be analyzed to finally proof the performance of the method. For this purpose, only human ECGs showing calcium concentration changes of more than 0.2 mmol/l would be of values since effects can vary among species.^52^

### B. Manual and semi-automatic approaches

Early approaches to reconstruct concentrations or diagnose potassium disorders were mostly based on manual evaluations of the ECG. Frohnert *et al.*^69^ evaluated ECG changes in 16 patients during HD. Systematically, they measured RR, PR, QU, and QT intervals, T and P wave duration and amplitudes, the amplitude of S, R, U waves, and T_on_−T_peak_ times, ST change, and T_75%_ times (i.e., the time point of 75% increase/decrease in T wave amplitude). They derived a formula for the calculation of the potassium concentrations from the T wave amplitude and T maximum time. This was presumably the first attempt to systematically estimate plasma potassium concentrations based on ECG measurements, however, with manual feature determination and without performance evaluation. The model was built with all the available data, i.e., no independent validation was performed.

Another early study was published by Johansson and Larsson analyzing two ECG features for diagnosing hypokalemia.^70^ They identified the sum of ST depression and U wave amplitude in II and V3 as the most relevant features in a cohort of 22 hypokalemic patients. Interestingly, a correction by subtraction of values during normokalemia of the respective patient was performed to account for inter-patient variability. Accurate prediction of mild hypokalemia (2.7–3.4 mmol/l) appeared impossible with the proposed method. In this early work, the need—at least for the chosen feature combination—for a patient-specific model was already visible. This is mostly not considered, especially in the studies discussed in Sec. II.

Wrenn *et al.* evaluated the ability of physicians to detect hyperkalemia just from the ECG.^71^ In their study comprising 220 patients, two annotators were involved, and sensitivity, specificity, and positive and negative predictive values (PPV/NPV) were computed. The results (best result for each parameter separately) were a sensitivity of 0.43, a PPV of 0.65, a specificity of 0.86, and a NPV of 0.69. The calculation of those standard classification performance indices is notable and generally boosts the impact of a study since such performance parameters make studies and methods comparable.

Very similar to the study by Wrenn *et al.* and aiming at expanding the work of Frohnert *et al.* to non-HD patients, Velagapudi *et al.* conducted a study trying to diagnose hyperkalemic patients.^72^ They used the T wave slope and T wave and QRS duration to build a regression model (coefficients were provided) for potassium concentration estimation. Features were determined manually in 84 patients (236 ECGs) for training and 23 patients (97 ECGs) for testing. Receiver operating characteristic (ROC) curve analysis revealed a maximum sensitivity (74%) and specificity (76%) product at 5.75 mmol/l, and the equilibrium point was at 5.74 mmol/l (both sensitivity and specificity 74%). Interestingly, this decision threshold is different from the established intervals for normo- and hyperkalemia (Sec. IV C, Table I). The area under the ROC curve (AUC) was 0.783. However, the authors give two sets of performance values for the test set (they call it the validation set). It seems that the first mentioned set of values is the ones from the training set (compare paper's supplement^72^).

**TABLE I.** Automatic classification methods for dyskalemia. As the methods by Galloway were evaluated on three different datasets from three different hospitals, intervals of the respective evaluation method are given. Furthermore, they tuned classification thresholds by two different approaches. First, they chose the equilibrium point of sensitivity and specificity (lines three and four). The evaluation with the threshold determined at a sensitivity of 0.9 is denoted by an S in the first column. The different classification tasks (CT) are given by: K ${↑}^{a}$ hyperkalemia (>5.3 mmol/l), K ${↑}^{b}$ hyperkalemia (>5.5 mmol/l), and K↓ hypokalemia (<3.5 mmol/l). ANN: artificial neural network, CNN: convolutional neural network, accuracy in %, Se: sensitivity in %, Sp: specificity in %, PPV/NPV: positive/negative predictive value in %, ROC-AUC: area under receiver operating characteristic curve, N_*Pat*_: number of patients, and n/a: not available/given.

Work	CT	Lead (s)	Features	Model	Accuracy	Se	Sp	PPV	NPV	ROC-AUC	N_Pat_
Wu *et al.*^74^	K ${↑}^{a}$	12-Lead	*T_A_*, *P_A_*, PR,	ANN	62.5	60	65	n/a	n/a	n/a	50
			QTc, QRS_*w*_								
Tzeng *et al.*^75^	K ${↑}^{a}$	12-Lead	*T_vol_*, PR,	K-means	n/a	85	79	n/a	n/a	n/a	97
			QT, QRS_*w*_								
Galloway *et al.*^14^	K ${↑}^{b}$	I, II	CNN	CNN	76.1–80.4	78.1–80.5	75.2–81.3	13.8–18.1	97.6–98.5	0.85–0.88	61 965
Galloway *et al.*^14^	K ${↑}^{b}$	I, II, V3, V5	CNN	CNN	77.4–82.6	81.3–84.0	77.1–84.2	11.0–15.4	98.9–99.4	0.88–0.90	61 965
Galloway *et al.*^14,*S*^	K ${↑}^{b}$	I, II	CNN	CNN	57.8–64.2	88.9–91.3	54.7–63.2	6.0–9.2	99.0–99.6	0.85–0.88	61 965
Galloway *et al.*^14,*S*^	K ${↑}^{b}$	I, II, V3, V5	CNN	CNN	63.9–69.0	89.3–92.6	60.3–70.0	7.2–10.5	99.4–99.6	0.88–0.90	61 965
Lin *et al.*^15^	K ${↑}^{b}$	12-Lead	CNN	CNN	n/a	50.8	96.0	26.9	98.5	0.91	40 180
Lin *et al.*^15^	K↓	12-Lead	CNN	CNN	n/a	50.7	81.6	44.7	85.0	0.75	40 180

Regolisti *et al.* utilized manually determined ECG features and used them to quantify serum potassium levels and to classify hyperkalemic ECGs.^73^ Using the Bayesian information criterion and leave-one-out cross-validation, they selected two out of 28 categorical and continuous features. Some were evaluated subjectively, like the peakedness of the T wave. The root mean squared error was 0.96 mmol/l using linear regression of T wave amplitude and a categorical offset term in the case of the use of diuretics. Logistic regression was used to classify hyperkalemia with the same inputs as before. They achieved a ROC-AUC of 0.74.

In this section, we gave an overview on manual and semi-automatic approaches for the detection of concentration disorders with the ECG. Over time, standard classification benchmark parameters were also applied in the clinical studies. As an example, the highest sensitivity among the presented studies was 0.74 with a corresponding specificity of 0.76 and a ROC-AUC of 0.783.^72^ Using these measures makes the studies comparable to the ones presented in Sec. IV C. We will see that results presented here are already in the range of the studies utilizing automatic approaches (Table I).

### C. Automatic approaches

**FIG. 3.**
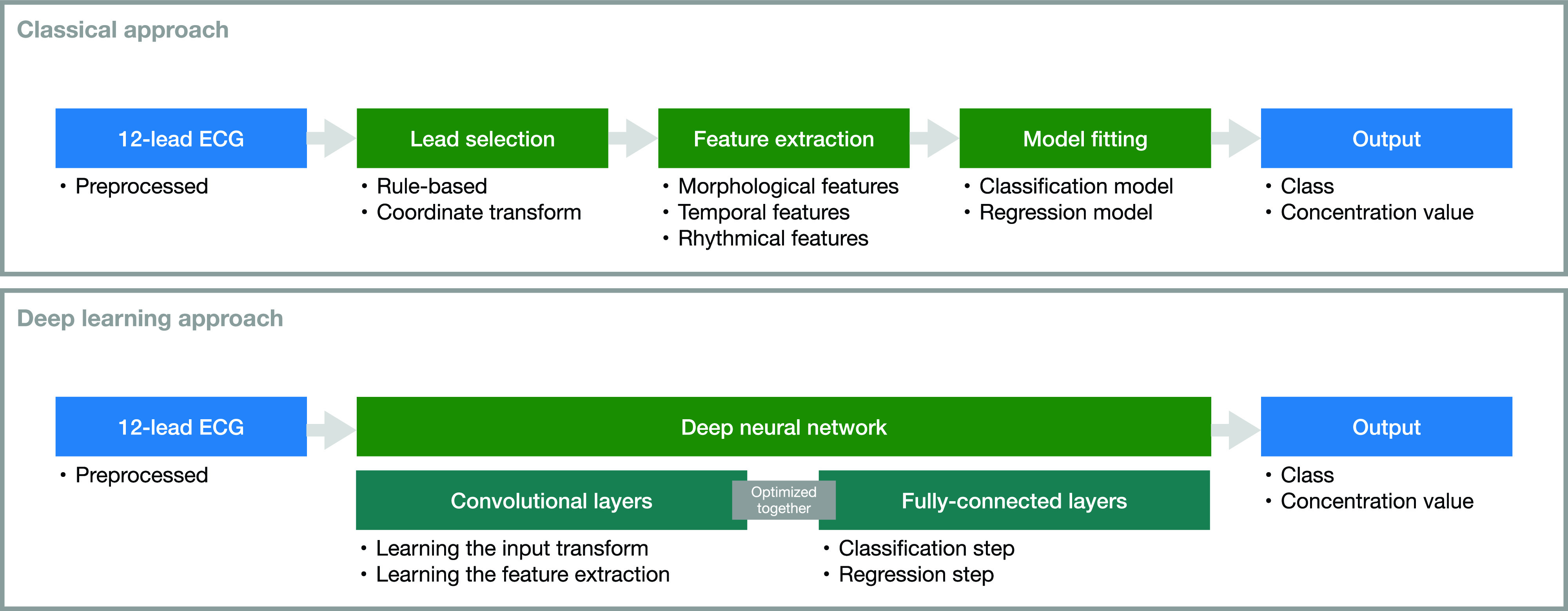
Overview of the two approaches for automatic concentration estimation found in the literature. In contrast to the classical approach where lead selection, feature selection, and model fitting are separated, in the deep learning approach, they are inherently integrated.

The next step is of course automatic diagnosis (classification, Table I) and quantification (regression, Table II) of electrolyte disorders. The two main approaches for solving the problem are summarized in Fig. 3. We can distinguish between methods relying on hand-crafted features and subsequent model creation on the one hand and deep learning methods where feature extraction is inherently integrated in the model creation on the other hand. Compared to the approaches in the last section, works in this section do not rely on manual feature extraction. In 2003, Wu *et al.* used a two-stage artificial neural network for hyperkalemia classification.^74^ The P wave amplitude and duration in lead II, PR interval, QTc, and QRS complex width were the inputs for the first stage and T wave amplitudes and widths from V1 to V6 for the second stage. Thus, a total of 16 features was used and yielded an accuracy of 65.5%, a sensitivity of 60%, and a specificity of 65% in a patient cohort with 30 normokalemic and 30 hyperkalemic patients. However, no information regarding the validation technique is provided, and so overfitting cannot be excluded. In particular, the rather small number of samples (patients) used with 16 features paired with a complex model (in total 200 hidden neurons in the networks) is likely to be prone to overfitting.^76^ Although the given sensitivity is better than that in the study by Wrenn *et al.*,^71^ specificity is worse, which could be a result of overfitting. In a follow-up study, Tzeng *et al.* decreased the number of used features and also chose a less complex model.^75^ 97 cases, of which 41 were hyperkalemic, were used to train a two-stage k-mean classifier. Two T wave volume features obtained from limb and chest leads were fed to the first stage and PR interval and QT interval and QRS complex width to the second. Interestingly, the classification was based on four classes with three of them representing hyperkalemia and one representing normokalemia. This might underline the different phenotypes of hyperkalemia since it was not possible to aggregate the hyperkalemia class clusters in the feature space to one connected cluster. The sensitivity increased compared to Wu *et al.*^74^ to 0.85 and specificity to 0.79. Again, no information regarding cross-validation to prevent overfitting was given. Nevertheless, model complexity was decreased, which is always recommended for small sample sizes.^76,77^ In contrast to the aforementioned works, the studies by Corsi and Severi *et al.* systematically evaluated the estimation of potassium concentration values instead of classifying the disorder.^13,78–80^ As the ECG feature, the ratio between the T wave downslope and amplitude was used. This feature was calculated from the two most significant PCA eigenleads of the 12-lead ECG using a template of the T wave computed on each two-minute window. Additionally, after building a general model, patient-specific bias correction was introduced using the first and last measurement of the first session of a specific patient. The regression models were polynomials of first order^78^ and second order.^13,79,80^ The latter technique was validated in a cohort of 45 patients (128 HD sessions) using leave-one-patient-out cross-validation, preventing overfitting and yielding an error of −0.09 ± 0.59 mmol/l (mean absolute error of 0.46 ± 0.39 mmol/l).^13^ In a parallel work, Attia *et al.* used a slightly modified approach.^81^ First, they changed the feature to the T downslope divided by the square root of T amplitude, while also the feature calculation was adjusted: templates of a 72 second ECG snippet were used for feature estimation and an additional Kalman filter was implemented to attenuate abrupt feature changes that cannot be related to a potassium change. They did not use the PCA of a 12-lead ECG signal but selected the lead with the highest T wave amplitude from V4 to V6. For regression, a first order polynomial was fitted. Second, for patient-specific calibration, they did not build a general model and added a patient-specific bias term. Instead, they built individual linear models with the chosen feature in the first session and applied them to the other sessions of an individual patient. This yielded a mean absolute error of 0.36 ± 0.34 mmol/l in their cohort of 26 patients. Moreover, they compared this result with the general model, fitting the model with a training (26 patients, estimation error 0.44 ± 0.47 mmol/l) and a validation cohort (19 patients, estimation error 0.5 ± 0.42 mmol/l). The proposed method was also applied to data from a handheld device by the same group.^82^ This time, lead selection was not necessary anymore. 21 HD patients were screened using the ECG captured using a commercially available ECG electrode system measuring between two fingertips of both hands. This setup yielded an error of 0.38 ± 0.32 mmol/l using patient-specific models, which is in the range of the performance of the approach using a standard 12-lead ECG. Beyond the extraction of hand-crafted features for training a method, convolutional neural networks (CNNs) learn the feature extraction in addition to the actual classification or regression task. The approach relies on the network identifying the best features itself.^83^ This is usually achieved through a serial and parallel combination of 1D, 2D, or 3D filters (convolutions) on 1D, 2D, or 3D input data. The structure of this convolutional network can be optimized regarding the given task. In the example of ECG-based concentration estimation, a 2D input could consist of several ECG leads with the lead number forming one dimension and the time forming the other. The image is processed in the filtering layers yielding intermediate outputs. Finally, all filtering results are connected within a final fully connected layer delivering the classification or regression result. This fully connected layer is optimized together with all the filtering kernels in the convolutional layers delivering an optimal result for the desired application. This approach, however, is often criticized for the non-transparency since, on the one hand, the extracted features might not be interpretable for humans and, on the other hand, the huge number of layers and features might not allow an interpretation. This setup is often referred to as deep neural network or more commonly as a deep learning method/network. Galloway *et al.* propose a deep learning method for the classification of hyperkalemia.^14^ Two models comprising ten convolutional layers for feature extraction and one fully connected layer were trained with ten second ECGs using leads I and II, or I, II, V3, and V5. Data used for training included approximately 1.5 × 10^6^ ECGs out of which approximately 2% were from hyperkalemic patients (potassium concentration ≥5.5 mmol/l, at least one blood-test was available within twelve hours before or after the ECG measurement). Since neural networks are fuzzy classifiers, ROC curves could be obtained for different classification thresholds. A common point to select is the equilibrium of sensitivity and specificity. The authors evaluated the proposed algorithm with data from three different centers showing that accuracy for two lead evaluation was between 76.1% and 80.4%, sensitivity between 78.1% and 80.5%, and specificity between 75.2% and 81.3%. The ROC-AUC was 0.883. The four lead approach improved results by 1%–2%, and ROC-AUC was 0.901. To use this method as a screening method, the authors proposed to select the point in the ROC curve showing a 90% sensitivity. This led to false negative rates of 0.3%–0.6% for the two lead approach and 0.3%–0.4% for the four lead network. In the supplement, the authors address the transparency of the algorithm. They applied feature visualization techniques to trace back possible patterns that cause the network as a fuzzy classifier to predict a high likelihood for hyperkalemia. They show three example beats in the supplement of their work, stating that one of them looks very atypical for a hyperkalemic ECG beat. The authors conclude that the deep learning method recognized additional morphological features being relevant for the classification, which are not apparent on visual screening.

**TABLE II.** Automatic regression methods for the potassium concentration. CNN: convolutional neural network, $T_{S/A}$: T downslope divided by T amplitude $T_{S/\sqrt{A}}$: T downslope divided by the square root of T amplitude, result: mean ± standard deviation of signed errors in mmol/l, result (abs): mean ± standard deviation of absolute values of errors in mmol/l, N_*Pat*_: number of patients, N_*Sess*_: number of HD sessions, n/r: not relevant if data were not from HD sessions, and n/a: not available/given.

Work	Lead (s)	Features	Model	Result (mmol/l)	Result (abs) (mmol/l)	Dataset (mmol/l)	N_*Pat*_	N_*Sess*_
Corsi *et al.*^13^	PCA	$T_{S/A}$	Polynomial second order	−0.09 ± 0.59	0.46 ± 0.39	n/a	45	128
Attia *et al.*^81^ personalized	V3−V5	$T_{S/\sqrt{A}}$	Polynomial first order	n/a	0.36 ± 0.34	4.2 ± 0.95	26	113
Attia *et al.*^81^ global	V3–V5	$T_{S/\sqrt{A}}$	Polynomial first order	n/a	0.50 ± 0.42	3.9 ± 0.8	26	113
Yasin *et al.*^82^	I	$T_{S/\sqrt{A}}$	Polynomial first order	n/a	0.38 ± 0.32	4.3 ± 0.8	18	n/a
Lin *et al.*^15^	12-Lead	CNN	CNN	n/a	0.53 ± n/a	n/a	40 180	n/r

**FIG. 4.**
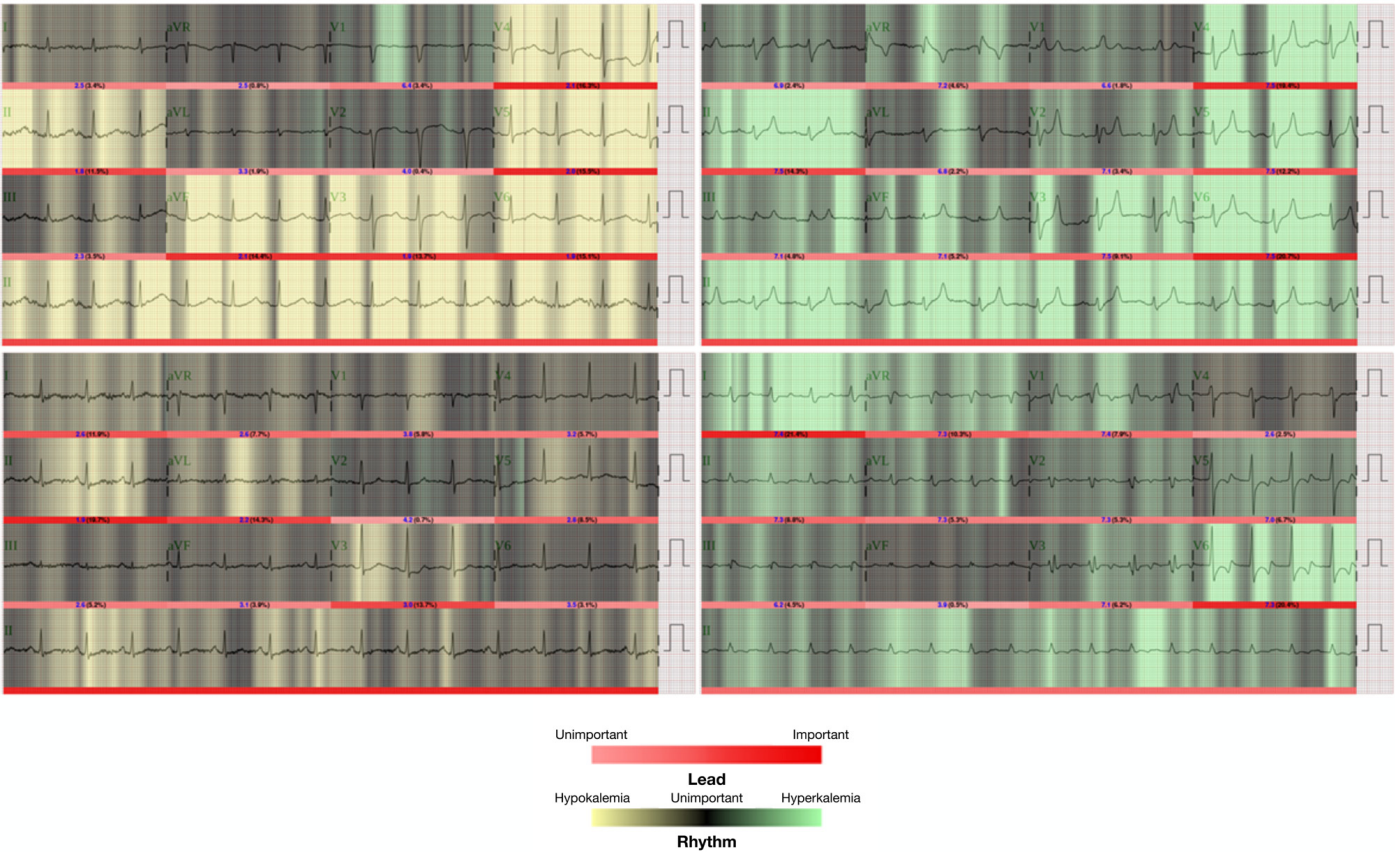
Explainability approach by visualization of parts of the input ECG being relevant for the classification and quantification of the potassium disorder. Important/unimportant parts of the ECG traces are visualized with bright and dark red bars under the traces. The rhythm classification is shown with black/greed/yellow backgrounds.^15^ Reproduced with permission from Lin *et al.*, JMIR Med. Inf. **8**(3), e15931 (2020). Copyright 2020, Authors licensed under a Creative Commons Attribution (CC BY) license.

The latest work was published in 2020 by Lin *et al.* and goes one step further.^15^ They propose a deep learning model, called ECG12Net comprising 80 convolutional layers and extracting 864 meta-features from the standard 12-lead ECG, aiming to diagnose dyskalemia. The model was based on 66 321 ECGs with a potassium measurement within one hour before or after ECG recording. After training with 32 176 ECGs, 8004 new samples were used for testing the method: the hypokalemia detection sensitivity was 50.7%, specificity 81.6%, positive predictive value 44.7%, and negative predictive value 85.0%, whereas the values for hyperkalemia were 50.8%, 96.0%, 26.9%, and 98.5%, respectively. The ROC-AUC values for hypokalemia detection (against others) were 0.75, and for hyperkalemia, it was 0.91. Adding a feed forward network fed with features calculated using a commercially available ECG device (heart rate, PR, QRS, QT interval, QTc, P wave axis, RS wave axis, and T wave axis) did not improve performance. Apart from the classification, a mean absolute error of 0.531 mmol/l is reported for regression (no standard deviation given; nevertheless, the 95% confidence interval is reported to be 0.523–0.539 mmol/l). In the work of Lin *et al.*, data from three emergency physicians and three cardiologists of different experience levels to classify 300 ECGs from the test data partition are compared. While the cardiologists showed similar performance compared to other studies, the proposed ECG12Net outperformed even the most experienced physicians regarding almost all performance parameters. Apart from the estimation method and the performance measures, the authors provide several further interesting points in their study. First, they visualized the parts in an ECG trace being relevant for the classification results (Fig. 4). This is interesting regarding two points: first, the importance of the ECG wave properties is visualized. Here, some single leads are not/less of interest, but others are more/highly relevant. All waveforms seem to deliver information used for the classification; nevertheless, T waves seem to be most frequently marked as important. Short-term rhythmical properties could be of relevance, too, since the input of the network was always several beats. The second important point connected with this visualization approach addresses the widely discussed topic of artificial intelligence explainability.^84,85^ This procedure is a promising step toward opening the “black box” of deep learning and helping to establish such a method in clinical practice. Furthermore, the authors provide a lot of relevant information regarding the network, its training, additionally tested methods, and evaluations in the supplement of their publication. The issue of an unequally distributed dataset and its handling was addressed as well.

From the two latter studies, both utilizing convolutional neural networks, one can appreciate the potential of automatic feature extraction. Regarding the dyskalemia classification accuracy, the methods outperform those using hand-crafted features as input regarding most of the performance parameters even when using only two Einthoven leads (compare Table I). However, the diagnostic performance of a concentration estimation is interesting and relevant as well. Although Lin *et al.* provide a mean absolute error, they do not report variability of the estimation errors. Thus, a comparison is hardly possible from this aspect. Nevertheless, from what we know, the performance is in the upper range of errors compared to the methods with hand-crafted features (Table II). However, the model by Lin *et al.* does not need to be individually adjusted for each patient as, e.g., the approach by Corsi *et al.*^13^

**FIG. 5.**
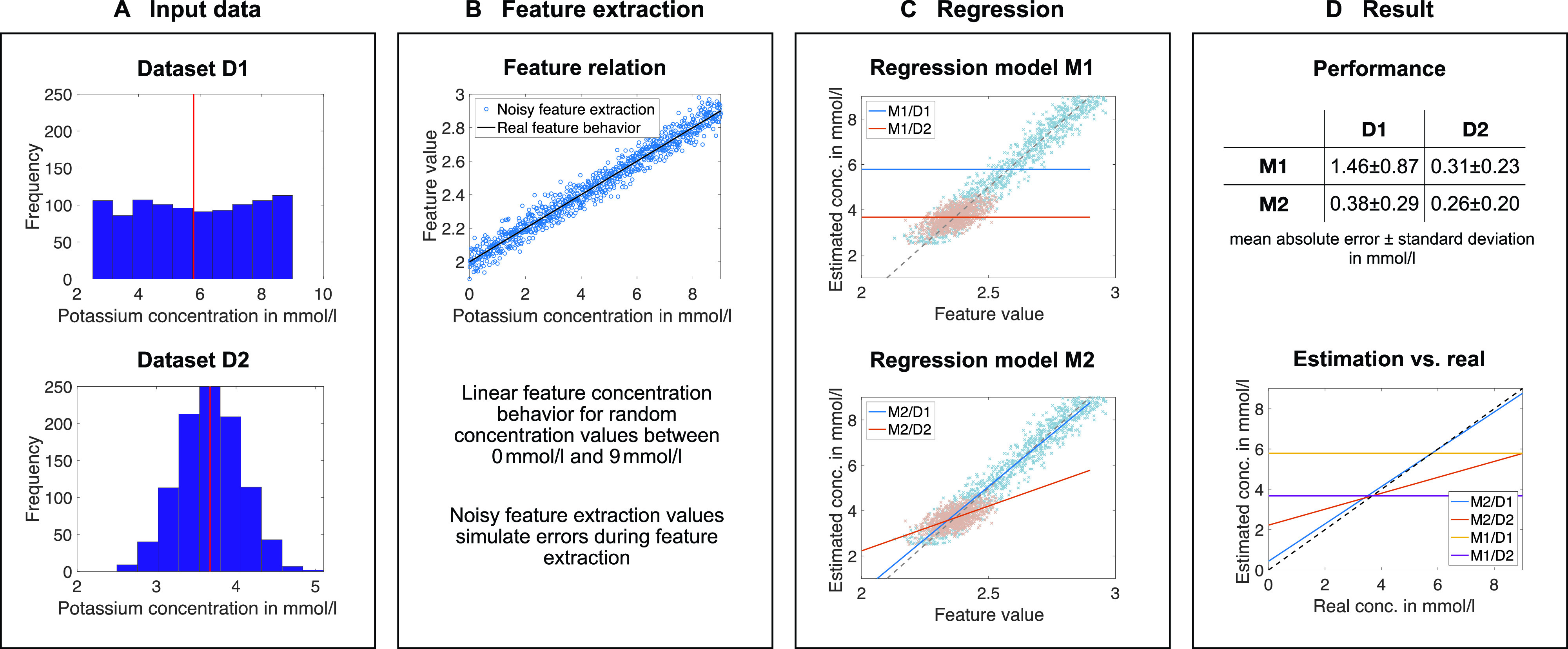
An example to illustrate the problem of using mean and standard deviation as sole performance parameters. (a) Two exemplary datasets D1 and D2 with a uniform and normal distribution, respectively. (b) An exemplary linear feature concentration dependence. For the subsequent fitting, feature values were randomly distorted by up to 5.5%. (c) The result of two regression methods. Method M1 is a fit with a constant minimizing the error, and model M2 is a linear fit in the least squares sense. The light red (dataset D2) and light blue (dataset D1) point clouds visualize the noisy feature inputs for the fitting yielding the models in red and blue, respectively. The dashed line is the real noise-free relation. (d) Results of both methods on both datasets. Although the combination of M2/D1 reconstructs the underlying dependency over the whole interval best, it is outperformed by the combination M2/D2 and M1/D2 when considering the mean absolute error. Within a dataset, the linear model (M2) always outperforms the constant (M1) as expected.

**FIG. 6.**
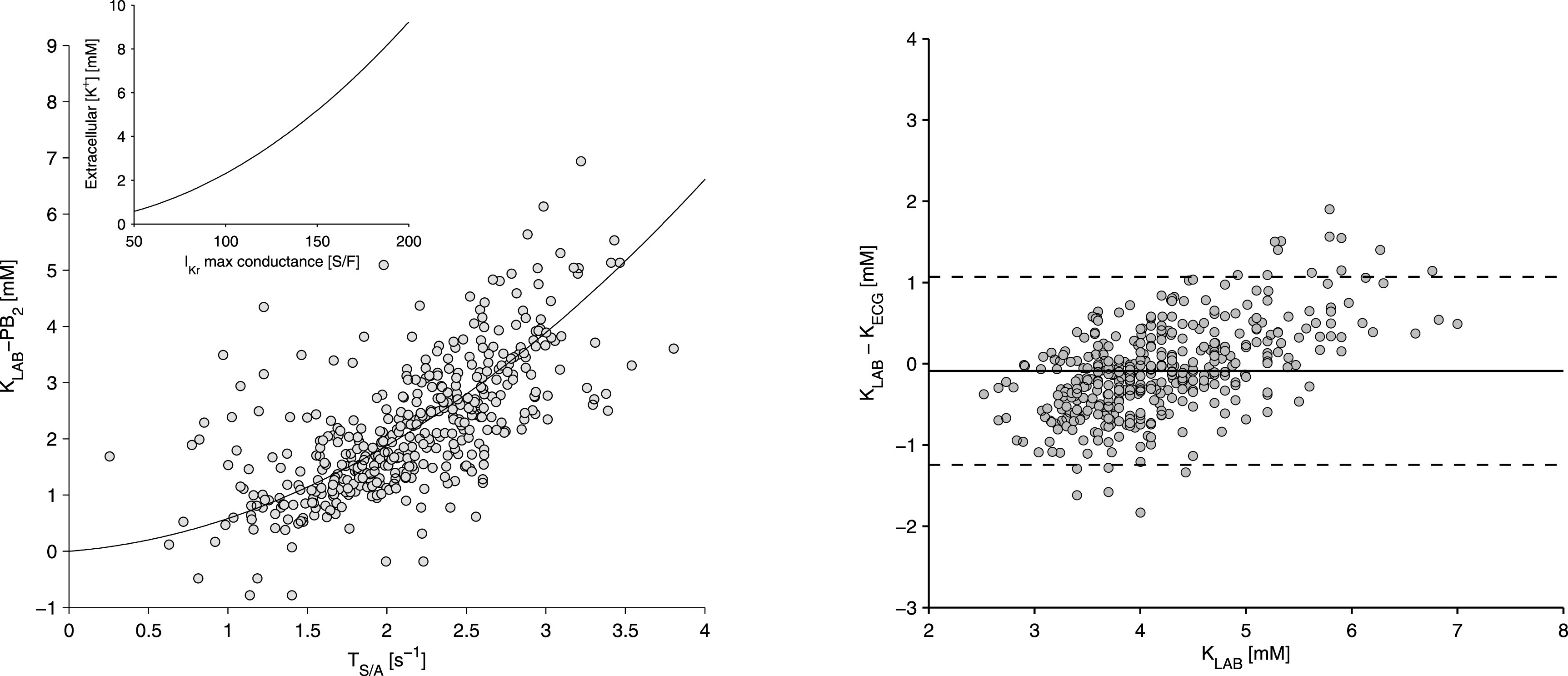
Potassium concentration estimation results by Corsi *et al.*^13^ Left: relation of the selected feature T wave downslope divided by T amplitude and the potassium blood measurement including a patient specific bias. Right: Bland–Altman plot of the estimation error. It is apparent that the method does not perform well for higher concentrations. Reproduced with permission from Corsi *et al.*, Sci. Rep. **7**, 42492 (2017). Copyright 2017, Authors licensed under a Creative Commons Attribution (CC BY) license.

We generally encourage the community to report the frequently used standard performance measures (for concentration estimation, these are mean values and standard deviations of the errors and absolute errors; for classification, these are sensitivity, specificity, negative/positive predictive value, AUC-ROC, and accuracy). Additionally, we encourage providing additional measures like confidence intervals, interquartile ranges, F-values, and, most importantly, detailed information on the distribution of concentration values and pathologies in the dataset. Of course, the optimal condition would be to have open datasets to compare methods. As this need is not met at the moment, the least we can do is providing as much information on the dataset as possible. The significance of this fact is exemplarily shown in Fig. 5: depending on the underlying distribution of potassium concentrations in the dataset, different fits of different qualities are achieved. However, it is shown that the performance measures do not reflect the actual quality of the fit. From this example, we can clearly conclude that first, a comparison is hardly possible without knowing the concentration distribution in the dataset. Second, only a standardized dataset enables reliable comparisons of different methods. Without the latter, the comparison of the methods shown in Tables I and II is complicated. Apart from the used datasets, e.g., Bland–Altman plots can help to assess the model's capability to estimate extremely high or low concentrations. The Bland–Altman plot in the study by Corsi *et al.*^13^ (Fig. 6) implies that the chosen model and feature combination were less accurate for extremely high or low concentrations. This could be related to the fact that the concentration distribution in the dataset was not considered during model fitting. Nevertheless, we want to emphasize that this can be legitimated if one wants to find the model being most suitable for the majority of concentration values, i.e., being more accurate for cases of mild hyper/hypokalemia and normokalemia at the expense of worse estimation for severe dyskalemia. In contrast, if one wants to detect higher values with an increased accuracy, either errors should be weighted in the fitting process based on the underlying distribution (as exemplarily performed in Ref. 86), or equally distributed concentration values in the training dataset are required. Only the mean and standard deviation values of the concentration distribution in the dataset or of the estimation errors do not provide enough information to setup a nearly comparable setting. However, this is important to really be able to evaluate a newly proposed concentration estimation approach and to be able to benchmark it with others.

## V. CURRENT STATE AND FUTURE STEPS

At the beginning of this paper, we discussed the relationship between clinical observations and manual ECG assessment, an approach that rather discourages one to use the ECG as a diagnostic tool for the estimation of plasma electrolyte concentrations. Results from modeling studies, on the other hand, contradict this conclusion and raise optimism for the applicability of ECG-based concentration estimation. Finally, the most important argument for concentration estimation with the ECG is the successful application of different approaches using a semi- or fully automatic reconstruction. Over time, they gained more and more precision dispelling the aforementioned doubts. The fact that automatic concentration estimation—either as classification or regression—yields results that are not only far away from chance but also showing accuracies for hyperkalemia detection of up to 82.6%,^14^ a maximum sensitivity above 92%,^14^ and a specificity of 96%^15^ (Table I), outperforming detection rates by physicians found in the presented works in Sec. IV B, which only underline this. Nevertheless, in the current state, we have automatic methods showing very promising results but still entailing many open questions. To tackle those, we see the urgent need for closer collaboration between clinicians and engineers to overcome open questions (e.g., about the compensation of cross-dependencies) and make further progress in this challenging field.

From the clinical side, we encourage to provide data and clinical experience as well as, in a future step, the acceptance for implementing new technologies in the clinical routine to build up a feedback loop to the engineers being the developers of the methods.

Engineers are in need to provide transparent and reproducible fully or semi-automatic algorithms to be used in a clinical environment (e.g., for feature extraction to overcome subjectivity in feature quantification and to increase precision of the resulting features that are sometimes still measured on paper). We want to suggest publishing results always together with data to foster comparability. A possible procedure including the documentation of parameters used during model creation and exact dataset properties was spearheaded by Lin *et al.* in the supplement.^15^ In addition, trained models could be provided to be able to compare the performance of new methods with those already existing. The same holds for the data used for evaluation. Furthermore, engineers should provide simulation models ready to contribute to answering clinical questions regarding the pathophysiological mechanisms that are not fully understood mechanistically. Having facilitated the latter, feature selection for the application in reconstruction techniques becomes easier as it could be more obvious what to look for. On the other hand, and not needing features, deep learning methods were shown to be beneficial for reconstructing the ion concentrations. This technique should further be exploited to find descriptive and interpretative features. A backtracing to the actual relevant changes in the ECG was done by Lin *et al.*^15^ and can be extended and further exploited using further explainability techniques.^84,85^ Moreover, the inclusion of features from rhythmical parameters as HRV could be fruitful for deep learning and the classical approaches since they are mostly utilizing short ECG segments neglecting possibly relevant long-term changes. Also, features from model fitting approaches seem to enable a more robust feature extraction and, therefore, more reliable results. Methods are already available, but their potential for concentration estimation still needs to be evaluated.

Joint forces are needed to find the exact reasons for current algorithms to fail (see the errors in Tables I and II). This could be caused by patient- or situation-specific confounding factors that were not accounted for, such as the amount of extracellular fluid as an important factor for amplitude changes,^87^ long QT2 syndrome affecting features used for concentration estimation,^13^ or other controlling mechanisms being more relevant for the ECG change than the actual concentration change. Large inter-patient variations that were already observed in an early study^88^ or inappropriate feature selection and determination could be reasons for suboptimal results as well. Further challenges comprise small sample sizes in most of the regression studies (Table II), biased datasets (ideally, an open standard dataset for training and evaluation is established), unknown confounding factors, and a benchmark method not always being as precise as expected.^16^ The latter fact is particularly problematic since we cannot be sure if the concentration estimation errors are caused by a methodological problem or by an inaccurate benchmark method.

Finally, there are two further topics staying in the background of current works and are worth to be researched more in-depth: automatic hypokalemia detection and plasma calcium concentration estimation. An automatic hypokalemia detection method was only proposed in one study^15^ and performed clearly worse than hyperkalemia detection (Table I). Nevertheless, hypokalemia was shown to be observed more frequently in hospitalized patients than hyperkalemia.^1^ The same holds for plasma calcium concentration estimation being at its beginning. Clinical data representing the relevant range of calcium concentration changes are still rare or not available. However, for developing and training an ECG-based calcium concentration evaluation method, these data are necessarily required. Such a tool could pave the way for elucidating the connection between the plasma calcium concentration and sudden cardiac deaths.

Many promising works from the clinical and the engineering domain were shown to contribute to a reliable ECG-based concentration estimation. The presented results not only support the feasibility of ECG-based concentration estimation but also are clearly heading toward a clinical application. If we strengthen collaboration between engineers and clinicians and perform goal-oriented experiments and studies, it is a clearly accomplishable goal to overcome current drawbacks and bring automatic concentration estimation to a level being applicable in clinical routine.

## Data Availability

Data sharing is not applicable to this article as no new data were created or analyzed in this study.
